# Bis(benzo[*h*]quinolin-10-olato-κ^2^ *N*,*O*)bromidomanganese(III)

**DOI:** 10.1107/S2414314620015709

**Published:** 2020-12-04

**Authors:** Veronica Papa, Anke Spannenberg, Matthias Beller, Kathrin Junge

**Affiliations:** a Leibniz-Institut für Katalyse e. V., Albert-Einstein-Str. 29a, 18059 Rostock, Germany; Vienna University of Technology, Austria

**Keywords:** crystal structure, manganese, benzo[*h*]quinolin-10-olate, π–π-stacking

## Abstract

The title compound, [MnBr(C_13_H_8_NO)_2_], consists of a manganese(III) atom, which is coordinated by one bromido and two benzo[*h*]quinolin-10-olato ligands. The Mn^III^ complex exhibits a distorted square-pyramidal coordination geometry with the Br ligand in the apical position. Neighbouring complexes are held together by π–π inter­actions and weak C—H⋯Br hydrogen bonds.

## Abstract 2

The manganese(III) atom of the title compound is coordinated by one bromide and two benzo[*h*]quinolin-10-olate ligands and exhibits a distorted square-pyramidal coordination environment.

## Graphical abstract

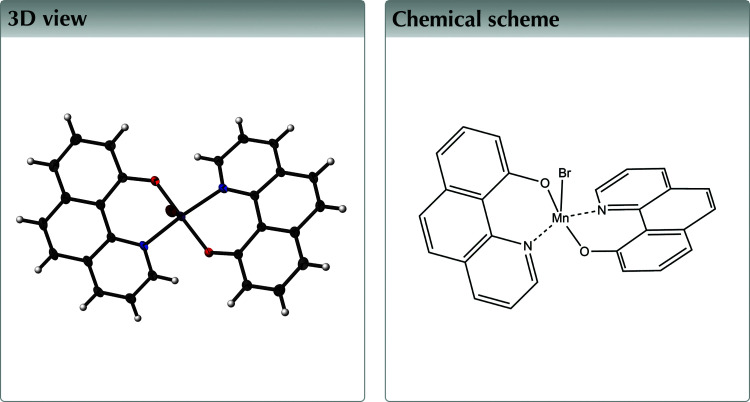

## Structure description

**Figure 1:**
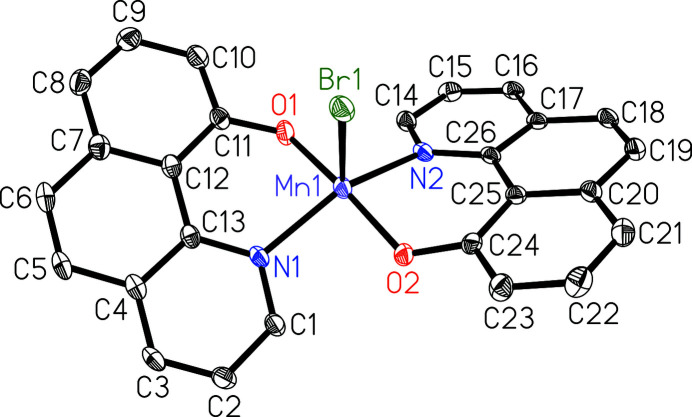
Mol­ecular structure of the title compound with atom labelling and displacement ellipsoids drawn at the 50% probability level.

Recently, we described the chemoselective reduction of quinolines and related *N*-heterocycles by mol­ecular hydrogen, using a simple Mn^I^ complex [Mn(CO)_5_Br] (Papa *et al.*, 2020 ▸). During the mechanistic studies of this catalytic reaction, several manganese compounds starting from [Mn(CO)_5_Br] and different *N*-heteroarenes were prepared and characterized by spectroscopic methods. In this context, the title compound was synthesized and structurally determined by single-crystal X-ray diffraction. The mol­ecular structure consists of a manganese(III) atom coordinated by one bromido and two bidentate benzo[*h*]quinolin-10-olato ligands (Fig. 1 ▸). The coordination environment around the Mn^III^ atom is best described as distorted square-pyramidal with the Br ligand in the apical position (*τ* = 0.35, with *τ* = 0 for an ideal square pyramid and *τ* = 1 for an ideal trigonal bypramid; Addison *et al.*, 1984 ▸). The deviation from planarity in the strained benzo[*h*]quinolin-10-olato ligands can be derived from the torsion angles N1—C13—C12—C11 of 10.0 (3)° and N2—C26—C25—C24 of 11.0 (3)°.

**Figure 2:**
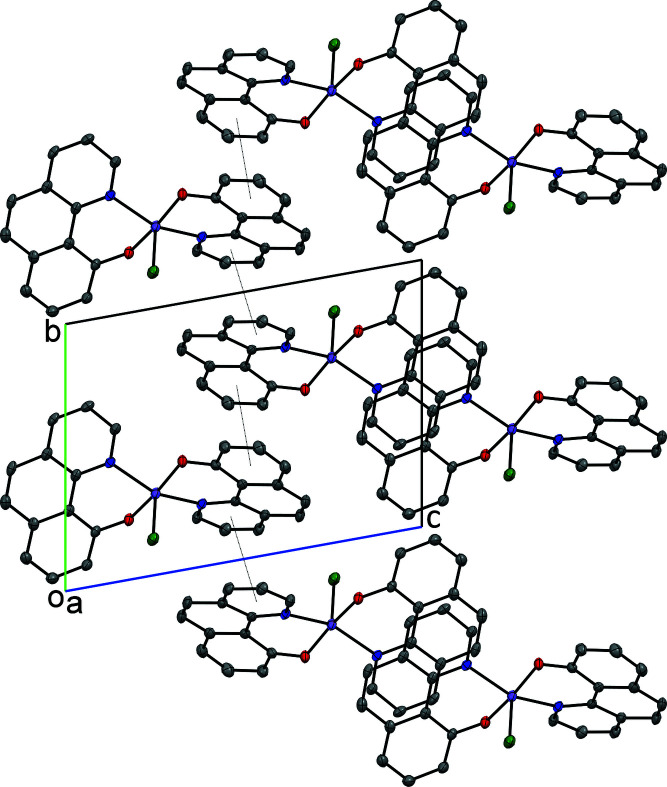
Packing diagram of the title compound along the *a* axis. Displacement ellipsoids are drawn at the 50% probability level. For clarity H atoms have been omitted. The alternating pattern of π–π stacking inter­actions between N1/C1–C4/C13 rings as well as between C7–C12 rings is shown with dotted lines.

In the crystal structure, π–π stacking inter­actions along the crystallographic *b* axis are observed between N1/C1–C4/C13 and between the C7–C12 rings, respectively (Fig. 2 ▸), with centroid-to-centroid distances *Cg*(N1/C1–C4/C13)⋯*Cg* ^i^(N1/C1–C4/C13) = 3.6804 (14) Å [symmetry code: (i) 1 − *x*, 2 − *y*, 1 − *z*], ring slippage = 1.42 Å, and *Cg*(C7–C12)⋯*Cg* ^ii^(C7–C12) = 3.6194 (16) Å [symmetry code: (ii) = 2 − *x*, 1 − *y*, 1 − *z*], ring slippage 1.33 Å. Neighbouring mol­ecules are also linked along the *a* axis by π–π stacking inter­actions between the aromatic ring systems N2/C14–C20/C25/C26 (Fig. 2 ▸) with centroid-to-centroid distances *Cg*(N2/C14–C20/C25/C26)⋯*Cg* ^iii^(N2/C14–C20/C25/C26) = 3.6310 (11) Å [symmetry code: (iii) =1 − *x*, 1 − *y*, 2 − *z*], ring slippage = 1.06 Å, and *Cg*(N2/C14–C20/C25/C26)⋯*Cg* ^iv^(N2/C14–C20/C25/C26) = 3.8165 (11) Å, [symmetry code: (iv) 2 − *x*, 1 − *y*, 2 − *z*], ring slippage 1.85 Å. Additionally, in the solid state weak inter­molecular C—H⋯Br inter­actions are observed (Table 1 ▸).

The crystal structure of a dimeric indium complex containing two benzo[*h*]quinolin-10-olato units has been reported by Wu *et al.* (1999 ▸). In addition, the crystal structure of 10-hy­droxy­benzo[*h*]quinoline has been described by Kubicki *et al.* (1995 ▸).

## Synthesis and crystallization

A mixture of solutions containing [Mn(CO)_5_Br] (0.02 mmol) and 10-hy­droxy­benzo[*h*]quinoline (0.5 mmol) in dry THF (2 ml) was stirred at 393 K for 18 h. The solvent was slowly removed in air giving dark-brown crystals after two weeks. Oxidation from Mn^I^ in the starting material to Mn^III^ in the product was mediated by atmospheric oxygen. Yield: 5.23 mg (50%); LCMS (*m*/z, pos): calculated for [C_26_H_16_BrMnN_2_O_2_] 521; found [*M* − Br]^+^ 443.

## Refinement

Crystal data, data collection and structure refinement details are summarized in Table 2 ▸. Seven outlier reflections were ignored during the refinement using the *OMIT* instruction.

## Supplementary Material

Crystal structure: contains datablock(s) I. DOI: 10.1107/S2414314620015709/wm4143sup1.cif

Structure factors: contains datablock(s) I. DOI: 10.1107/S2414314620015709/wm4143Isup2.hkl

CCDC reference: 2047278

Additional supporting information:  crystallographic information; 3D view; checkCIF report

## Figures and Tables

**Table 1:** Hydrogen-bond geometry (Å, °)

*D*—H⋯*A*	*D*—H	H⋯*A*	*D*⋯*A*	*D*—H⋯*A*
C1—H1⋯Br1^i^	0.95	3.03	3.674 (2)	126
C2—H2⋯Br1^i^	0.95	2.96	3.626 (3)	128

Symmetry code: (i) 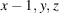 .

**Table 2:** Experimental details

Crystal data	
Chemical formula	[MnBr(C_13_H_8_NO)_2_]
*M*_r_	523.26
Crystal system, space group	Triclinic, *P* 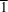
Temperature (K)	150
*a*, *b*, *c* (Å)	7.3303 (5), 10.5266 (7), 13.8432 (10)
α, β, γ (°)	76.9612 (18), 78.8220 (18), 72.0364 (18)
*V* (Å^3^)	980.97 (12)
*Z*	2
Radiation type	Mo *K*α
μ (mm^−1^)	2.74
Crystal size (mm)	0.41 × 0.23 × 0.13

Data collection	
Diffractometer	Bruker APEXII CCD
Absorption correction	Multi-scan (*SADABS*; Bruker, 2014 ▸)
*T*_min_, *T*_max_	0.40, 0.72
No. of measured, independent and observed [*I* > 2σ(*I*)] reflections	42556, 6093, 5110
*R*_int_	0.036
(sin θ/λ)_max_ (Å^−1^)	0.719

Refinement	
*R*[*F* ^2^ > 2σ(*F* ^2^)], *wR*(*F* ^2^), *S*	0.038, 0.114, 1.10
No. of reflections	6093
No. of parameters	289
H-atom treatment	H-atom parameters constrained
Δρ_max_, Δρ_min_ (e Å^−3^)	0.96, −0.44

Computer programs: *APEX2* (Bruker, 2014 ▸), *SAINT* (Bruker, 2013 ▸), *SHELXS97* (Sheldrick, 2008 ▸), *SHELXL2018/3* (Sheldrick, 2015 ▸), *XP* in *SHELXTL* (Sheldrick, 2015 ▸), *Mercury* (Macrae *et al.*, 2020 ▸) and *publCIF* (Westrip, 2010 ▸).

## full crystallographic data

### Crystal data

**Table:**

[MnBr(C_13_H_8_NO)_2_]	*Z* = 2
*M**_r_* = 523.26	*F*(000) = 524
Triclinic, *P*1	*D*_x_ = 1.771 Mg m^−^^3^
*a* = 7.3303 (5) Å	Mo *K*α radiation, λ = 0.71073 Å
*b* = 10.5266 (7) Å	Cell parameters from 9946 reflections
*c* = 13.8432 (10) Å	θ = 2.3–30.7°
α = 76.9612 (18)°	µ = 2.74 mm^−^^1^
β = 78.8220 (18)°	*T* = 150 K
γ = 72.0364 (18)°	Prism, brown
*V* = 980.97 (12) Å^3^	0.41 × 0.23 × 0.13 mm

### Data collection

**Table:**

Bruker APEXII CCD diffractometer	6093 independent reflections
Radiation source: fine-focus sealed tube	5110 reflections with *I* > 2σ(*I*)
Detector resolution: 8.3333 pixels mm^-1^	*R*_int_ = 0.036
ω scans	θ_max_ = 30.7°, θ_min_ = 2.1°
Absorption correction: multi-scan (*SADABS*; Bruker, 2014)	*h* = −10→10
*T*_min_ = 0.40, *T*_max_ = 0.72	*k* = −15→15
42556 measured reflections	*l* = −19→19

### Refinement

**Table:**

Refinement on *F*^2^	0 restraints
Least-squares matrix: full	Hydrogen site location: inferred from neighbouring sites
*R*[*F*^2^ > 2σ(*F*^2^)] = 0.038	H-atom parameters constrained
*wR*(*F*^2^) = 0.114	*w* = 1/[σ^2^(*F*_o_^2^) + (0.0649*P*)^2^ + 0.8212*P*] where *P* = (*F*_o_^2^ + 2*F*_c_^2^)/3
*S* = 1.10	(Δ/σ)_max_ = 0.001
6093 reflections	Δρ_max_ = 0.96 e Å^−^^3^
289 parameters	Δρ_min_ = −0.44 e Å^−^^3^

### Special details

**Table:**

Geometry. All esds (except the esd in the dihedral angle between two l.s. planes) are estimated using the full covariance matrix. The cell esds are taken into account individually in the estimation of esds in distances, angles and torsion angles; correlations between esds in cell parameters are only used when they are defined by crystal symmetry. An approximate (isotropic) treatment of cell esds is used for estimating esds involving l.s. planes.

### Fractional atomic coordinates and isotropic or equivalent isotropic displacement parameters (Å^2^)

**Table:**

	*x*	*y*	*z*	*U*_iso_*/*U*_eq_	
Br1	0.86544 (3)	0.86413 (2)	0.75405 (2)	0.02263 (8)	
C1	0.3671 (3)	0.8124 (2)	0.63030 (17)	0.0194 (4)	
H1	0.300222	0.800888	0.696278	0.023*	
C2	0.2593 (4)	0.8771 (3)	0.55156 (18)	0.0217 (5)	
H2	0.121977	0.905678	0.563390	0.026*	
C3	0.3551 (4)	0.8984 (3)	0.45723 (18)	0.0217 (5)	
H3	0.284870	0.945580	0.402862	0.026*	
C4	0.5574 (4)	0.8506 (2)	0.44051 (17)	0.0192 (4)	
C5	0.6605 (4)	0.8746 (3)	0.34209 (18)	0.0239 (5)	
H5	0.591514	0.924163	0.287706	0.029*	
C6	0.8552 (4)	0.8272 (3)	0.32630 (18)	0.0245 (5)	
H6	0.922096	0.848863	0.261538	0.029*	
C7	0.9628 (4)	0.7450 (2)	0.40491 (17)	0.0206 (4)	
C8	1.1609 (4)	0.6838 (3)	0.38329 (19)	0.0239 (5)	
H8	1.225262	0.704000	0.317627	0.029*	
C9	1.2636 (4)	0.5947 (3)	0.45581 (19)	0.0256 (5)	
H9	1.397247	0.551630	0.439627	0.031*	
C10	1.1717 (4)	0.5675 (3)	0.55334 (18)	0.0230 (5)	
H10	1.243939	0.506170	0.603124	0.028*	
C11	0.9763 (3)	0.6288 (2)	0.57866 (17)	0.0179 (4)	
C12	0.8661 (3)	0.7198 (2)	0.50432 (16)	0.0172 (4)	
C13	0.6599 (3)	0.7784 (2)	0.52252 (16)	0.0163 (4)	
C14	0.8677 (3)	0.4264 (2)	0.85999 (17)	0.0185 (4)	
H14	0.905836	0.409618	0.793397	0.022*	
C15	0.9360 (3)	0.3231 (2)	0.93801 (18)	0.0200 (4)	
H15	1.015414	0.236871	0.924909	0.024*	
C16	0.8865 (3)	0.3481 (2)	1.03367 (17)	0.0193 (4)	
H16	0.935733	0.280183	1.087564	0.023*	
C17	0.7636 (3)	0.4735 (2)	1.05221 (16)	0.0171 (4)	
C18	0.7158 (3)	0.5039 (3)	1.15135 (17)	0.0209 (4)	
H18	0.766023	0.437282	1.205665	0.025*	
C19	0.6004 (4)	0.6261 (3)	1.16818 (17)	0.0216 (4)	
H19	0.576532	0.646090	1.233820	0.026*	
C20	0.5124 (3)	0.7270 (2)	1.08935 (17)	0.0188 (4)	
C21	0.3829 (4)	0.8493 (3)	1.11064 (18)	0.0225 (5)	
H21	0.360503	0.868322	1.176527	0.027*	
C22	0.2875 (4)	0.9428 (3)	1.03660 (19)	0.0245 (5)	
H22	0.199403	1.025525	1.051751	0.029*	
C23	0.3201 (4)	0.9160 (2)	0.93963 (18)	0.0221 (5)	
H23	0.251384	0.979792	0.889550	0.027*	
C24	0.4518 (3)	0.7971 (2)	0.91527 (16)	0.0171 (4)	
C25	0.5548 (3)	0.7003 (2)	0.98981 (16)	0.0160 (4)	
C26	0.6900 (3)	0.5732 (2)	0.97060 (16)	0.0155 (4)	
Mn1	0.69313 (5)	0.69516 (4)	0.74703 (2)	0.01740 (9)	
N1	0.5603 (3)	0.76591 (19)	0.61740 (14)	0.0166 (3)	
N2	0.7511 (3)	0.54816 (19)	0.87462 (14)	0.0162 (3)	
O1	0.8970 (3)	0.59267 (18)	0.67213 (12)	0.0215 (3)	
O2	0.4684 (2)	0.77507 (17)	0.82180 (12)	0.0196 (3)	

### Atomic displacement parameters (Å^2^)

**Table:**

	*U*^11^	*U*^22^	*U*^33^	*U*^12^	*U*^13^	*U*^23^
Br1	0.02412 (13)	0.02379 (13)	0.01929 (12)	−0.00630 (9)	−0.00649 (9)	0.00014 (9)
C1	0.0198 (10)	0.0235 (11)	0.0154 (10)	−0.0071 (9)	−0.0062 (8)	0.0002 (8)
C2	0.0196 (11)	0.0251 (11)	0.0206 (11)	−0.0057 (9)	−0.0080 (9)	−0.0009 (9)
C3	0.0240 (11)	0.0238 (11)	0.0179 (10)	−0.0060 (9)	−0.0109 (9)	0.0014 (8)
C4	0.0260 (11)	0.0174 (10)	0.0149 (10)	−0.0062 (8)	−0.0074 (8)	−0.0002 (8)
C5	0.0315 (13)	0.0254 (11)	0.0140 (10)	−0.0077 (10)	−0.0073 (9)	0.0017 (9)
C6	0.0321 (13)	0.0264 (12)	0.0135 (10)	−0.0085 (10)	−0.0031 (9)	−0.0001 (9)
C7	0.0258 (11)	0.0211 (10)	0.0168 (10)	−0.0088 (9)	−0.0027 (9)	−0.0039 (8)
C8	0.0248 (12)	0.0286 (12)	0.0188 (11)	−0.0091 (10)	0.0004 (9)	−0.0058 (9)
C9	0.0205 (11)	0.0316 (13)	0.0232 (12)	−0.0049 (10)	0.0001 (9)	−0.0076 (10)
C10	0.0226 (11)	0.0250 (11)	0.0191 (11)	−0.0008 (9)	−0.0052 (9)	−0.0052 (9)
C11	0.0204 (10)	0.0177 (10)	0.0153 (9)	−0.0026 (8)	−0.0039 (8)	−0.0045 (8)
C12	0.0211 (10)	0.0150 (9)	0.0154 (10)	−0.0049 (8)	−0.0035 (8)	−0.0019 (7)
C13	0.0205 (10)	0.0144 (9)	0.0141 (9)	−0.0052 (8)	−0.0043 (8)	−0.0006 (7)
C14	0.0206 (10)	0.0173 (10)	0.0168 (10)	−0.0056 (8)	−0.0044 (8)	0.0006 (8)
C15	0.0185 (10)	0.0157 (10)	0.0230 (11)	−0.0042 (8)	−0.0044 (8)	0.0028 (8)
C16	0.0169 (10)	0.0203 (10)	0.0183 (10)	−0.0057 (8)	−0.0064 (8)	0.0055 (8)
C17	0.0143 (9)	0.0209 (10)	0.0159 (9)	−0.0074 (8)	−0.0044 (8)	0.0027 (8)
C18	0.0191 (10)	0.0284 (12)	0.0147 (10)	−0.0092 (9)	−0.0055 (8)	0.0036 (8)
C19	0.0202 (10)	0.0308 (12)	0.0143 (10)	−0.0086 (9)	−0.0031 (8)	−0.0022 (9)
C20	0.0184 (10)	0.0234 (11)	0.0164 (10)	−0.0083 (8)	−0.0037 (8)	−0.0026 (8)
C21	0.0251 (11)	0.0253 (11)	0.0184 (10)	−0.0088 (9)	−0.0006 (9)	−0.0056 (9)
C22	0.0277 (12)	0.0185 (11)	0.0233 (12)	−0.0034 (9)	0.0016 (9)	−0.0040 (9)
C23	0.0240 (11)	0.0183 (10)	0.0187 (11)	−0.0025 (9)	−0.0008 (9)	0.0009 (8)
C24	0.0156 (9)	0.0187 (10)	0.0149 (9)	−0.0049 (8)	−0.0008 (8)	0.0005 (8)
C25	0.0136 (9)	0.0184 (10)	0.0154 (9)	−0.0049 (8)	−0.0027 (7)	−0.0003 (8)
C26	0.0135 (9)	0.0194 (10)	0.0146 (9)	−0.0074 (8)	−0.0044 (7)	0.0011 (8)
Mn1	0.01791 (17)	0.01906 (17)	0.01235 (16)	−0.00190 (13)	−0.00333 (12)	−0.00042 (12)
N1	0.0190 (9)	0.0171 (8)	0.0132 (8)	−0.0050 (7)	−0.0057 (7)	0.0013 (6)
N2	0.0171 (8)	0.0163 (8)	0.0151 (8)	−0.0049 (7)	−0.0052 (7)	0.0002 (7)
O1	0.0232 (8)	0.0224 (8)	0.0126 (7)	0.0019 (6)	−0.0026 (6)	−0.0018 (6)
O2	0.0177 (7)	0.0226 (8)	0.0134 (7)	−0.0006 (6)	−0.0018 (6)	−0.0003 (6)

### Geometric parameters (Å, º)

**Table:**

Br1—Mn1	2.5060 (5)	C14—H14	0.9500
C1—N1	1.338 (3)	C15—C16	1.366 (3)
C1—C2	1.394 (3)	C15—H15	0.9500
C1—H1	0.9500	C16—C17	1.397 (3)
C2—C3	1.363 (3)	C16—H16	0.9500
C2—H2	0.9500	C17—C26	1.422 (3)
C3—C4	1.401 (3)	C17—C18	1.432 (3)
C3—H3	0.9500	C18—C19	1.346 (4)
C4—C13	1.421 (3)	C18—H18	0.9500
C4—C5	1.433 (3)	C19—C20	1.436 (3)
C5—C6	1.350 (4)	C19—H19	0.9500
C5—H5	0.9500	C20—C21	1.398 (3)
C6—C7	1.429 (3)	C20—C25	1.424 (3)
C6—H6	0.9500	C21—C22	1.381 (4)
C7—C8	1.398 (4)	C21—H21	0.9500
C7—C12	1.428 (3)	C22—C23	1.394 (4)
C8—C9	1.373 (4)	C22—H22	0.9500
C8—H8	0.9500	C23—C24	1.389 (3)
C9—C10	1.397 (4)	C23—H23	0.9500
C9—H9	0.9500	C24—O2	1.341 (3)
C10—C11	1.388 (3)	C24—C25	1.424 (3)
C10—H10	0.9500	C25—C26	1.444 (3)
C11—O1	1.334 (3)	C26—N2	1.375 (3)
C11—C12	1.425 (3)	Mn1—O2	1.8356 (17)
C12—C13	1.441 (3)	Mn1—O1	1.8389 (17)
C13—N1	1.373 (3)	Mn1—N2	2.0849 (19)
C14—N2	1.337 (3)	Mn1—N1	2.0858 (18)
C14—C15	1.394 (3)		
			
N1—C1—C2	123.1 (2)	C16—C17—C26	119.1 (2)
N1—C1—H1	118.4	C16—C17—C18	120.9 (2)
C2—C1—H1	118.4	C26—C17—C18	119.9 (2)
C3—C2—C1	118.6 (2)	C19—C18—C17	120.5 (2)
C3—C2—H2	120.7	C19—C18—H18	119.7
C1—C2—H2	120.7	C17—C18—H18	119.7
C2—C3—C4	120.0 (2)	C18—C19—C20	121.6 (2)
C2—C3—H3	120.0	C18—C19—H19	119.2
C4—C3—H3	120.0	C20—C19—H19	119.2
C3—C4—C13	119.2 (2)	C21—C20—C25	120.3 (2)
C3—C4—C5	120.6 (2)	C21—C20—C19	120.2 (2)
C13—C4—C5	120.1 (2)	C25—C20—C19	119.5 (2)
C6—C5—C4	120.3 (2)	C22—C21—C20	120.5 (2)
C6—C5—H5	119.8	C22—C21—H21	119.8
C4—C5—H5	119.8	C20—C21—H21	119.8
C5—C6—C7	121.4 (2)	C21—C22—C23	120.1 (2)
C5—C6—H6	119.3	C21—C22—H22	119.9
C7—C6—H6	119.3	C23—C22—H22	119.9
C8—C7—C12	120.1 (2)	C24—C23—C22	120.9 (2)
C8—C7—C6	119.8 (2)	C24—C23—H23	119.6
C12—C7—C6	120.0 (2)	C22—C23—H23	119.6
C9—C8—C7	120.8 (2)	O2—C24—C23	117.5 (2)
C9—C8—H8	119.6	O2—C24—C25	122.3 (2)
C7—C8—H8	119.6	C23—C24—C25	120.1 (2)
C8—C9—C10	120.0 (2)	C24—C25—C20	118.0 (2)
C8—C9—H9	120.0	C24—C25—C26	123.1 (2)
C10—C9—H9	120.0	C20—C25—C26	118.7 (2)
C11—C10—C9	121.0 (2)	N2—C26—C17	119.6 (2)
C11—C10—H10	119.5	N2—C26—C25	121.04 (19)
C9—C10—H10	119.5	C17—C26—C25	119.4 (2)
O1—C11—C10	117.4 (2)	O2—Mn1—O1	170.00 (9)
O1—C11—C12	122.6 (2)	O2—Mn1—N2	86.73 (7)
C10—C11—C12	119.9 (2)	O1—Mn1—N2	90.35 (8)
C11—C12—C7	118.1 (2)	O2—Mn1—N1	90.70 (8)
C11—C12—C13	123.2 (2)	O1—Mn1—N1	86.90 (8)
C7—C12—C13	118.5 (2)	N2—Mn1—N1	149.10 (8)
N1—C13—C4	119.3 (2)	O2—Mn1—Br1	95.03 (6)
N1—C13—C12	121.41 (19)	O1—Mn1—Br1	94.96 (6)
C4—C13—C12	119.3 (2)	N2—Mn1—Br1	104.39 (5)
N2—C14—C15	123.0 (2)	N1—Mn1—Br1	106.51 (6)
N2—C14—H14	118.5	C1—N1—C13	119.53 (19)
C15—C14—H14	118.5	C1—N1—Mn1	116.41 (15)
C16—C15—C14	118.7 (2)	C13—N1—Mn1	123.77 (15)
C16—C15—H15	120.6	C14—N2—C26	119.37 (19)
C14—C15—H15	120.6	C14—N2—Mn1	116.60 (15)
C15—C16—C17	120.0 (2)	C26—N2—Mn1	123.79 (15)
C15—C16—H16	120.0	C11—O1—Mn1	128.18 (15)
C17—C16—H16	120.0	C24—O2—Mn1	126.02 (15)
			
N1—C1—C2—C3	−2.4 (4)	C20—C21—C22—C23	−0.2 (4)
C1—C2—C3—C4	2.6 (4)	C21—C22—C23—C24	−1.6 (4)
C2—C3—C4—C13	0.9 (4)	C22—C23—C24—O2	176.7 (2)
C2—C3—C4—C5	−178.7 (2)	C22—C23—C24—C25	0.4 (4)
C3—C4—C5—C6	−179.4 (2)	O2—C24—C25—C20	−173.6 (2)
C13—C4—C5—C6	1.0 (4)	C23—C24—C25—C20	2.5 (3)
C4—C5—C6—C7	4.0 (4)	O2—C24—C25—C26	2.2 (3)
C5—C6—C7—C8	172.1 (3)	C23—C24—C25—C26	178.4 (2)
C5—C6—C7—C12	−4.7 (4)	C21—C20—C25—C24	−4.3 (3)
C12—C7—C8—C9	2.6 (4)	C19—C20—C25—C24	174.4 (2)
C6—C7—C8—C9	−174.1 (2)	C21—C20—C25—C26	179.7 (2)
C7—C8—C9—C10	−2.0 (4)	C19—C20—C25—C26	−1.6 (3)
C8—C9—C10—C11	0.3 (4)	C16—C17—C26—N2	−4.5 (3)
C9—C10—C11—O1	176.9 (2)	C18—C17—C26—N2	173.8 (2)
C9—C10—C11—C12	0.7 (4)	C16—C17—C26—C25	176.7 (2)
O1—C11—C12—C7	−176.1 (2)	C18—C17—C26—C25	−5.0 (3)
C10—C11—C12—C7	0.0 (3)	C24—C25—C26—N2	11.0 (3)
O1—C11—C12—C13	−1.5 (3)	C20—C25—C26—N2	−173.3 (2)
C10—C11—C12—C13	174.6 (2)	C24—C25—C26—C17	−170.3 (2)
C8—C7—C12—C11	−1.6 (3)	C20—C25—C26—C17	5.5 (3)
C6—C7—C12—C11	175.1 (2)	C2—C1—N1—C13	−1.6 (3)
C8—C7—C12—C13	−176.4 (2)	C2—C1—N1—Mn1	172.44 (19)
C6—C7—C12—C13	0.3 (3)	C4—C13—N1—C1	5.2 (3)
C3—C4—C13—N1	−4.9 (3)	C12—C13—N1—C1	−174.8 (2)
C5—C4—C13—N1	174.7 (2)	C4—C13—N1—Mn1	−168.38 (16)
C3—C4—C13—C12	175.1 (2)	C12—C13—N1—Mn1	11.6 (3)
C5—C4—C13—C12	−5.3 (3)	C15—C14—N2—C26	−1.8 (3)
C11—C12—C13—N1	10.0 (3)	C15—C14—N2—Mn1	172.88 (18)
C7—C12—C13—N1	−175.5 (2)	C17—C26—N2—C14	5.0 (3)
C11—C12—C13—C4	−170.0 (2)	C25—C26—N2—C14	−176.2 (2)
C7—C12—C13—C4	4.5 (3)	C17—C26—N2—Mn1	−169.30 (15)
N2—C14—C15—C16	−2.0 (4)	C25—C26—N2—Mn1	9.5 (3)
C14—C15—C16—C17	2.5 (3)	C10—C11—O1—Mn1	150.17 (19)
C15—C16—C17—C26	0.7 (3)	C12—C11—O1—Mn1	−33.7 (3)
C15—C16—C17—C18	−177.5 (2)	N2—Mn1—O1—C11	−169.3 (2)
C16—C17—C18—C19	178.6 (2)	N1—Mn1—O1—C11	41.5 (2)
C26—C17—C18—C19	0.4 (3)	Br1—Mn1—O1—C11	−64.8 (2)
C17—C18—C19—C20	3.6 (4)	C23—C24—O2—Mn1	143.34 (18)
C18—C19—C20—C21	175.7 (2)	C25—C24—O2—Mn1	−40.4 (3)
C18—C19—C20—C25	−3.0 (3)	N2—Mn1—O2—C24	45.79 (18)
C25—C20—C21—C22	3.2 (4)	N1—Mn1—O2—C24	−165.02 (18)
C19—C20—C21—C22	−175.5 (2)	Br1—Mn1—O2—C24	−58.38 (18)

### Hydrogen-bond geometry (Å, º)

**Table:**

*D*—H···*A*	*D*—H	H···*A*	*D*···*A*	*D*—H···*A*
C1—H1···Br1^i^	0.95	3.03	3.674 (2)	126
C2—H2···Br1^i^	0.95	2.96	3.626 (3)	128

Symmetry code: (i) *x*−1, *y*, *z*.
